# Editorial: Conversational AI

**DOI:** 10.3389/frai.2023.1203910

**Published:** 2023-05-10

**Authors:** Stephan Raaijmakers, Anita Cremers, Emiel Krahmer, Matthijs Westera

**Affiliations:** ^1^Leiden University Centre for Linguistics (LUCL), Leiden University, Leiden, Netherlands; ^2^Netherlands Organisation for Applied Scientific Research (TNO), Data Science Department, Amsterdam, Netherlands; ^3^Netherlands Organisation for Applied Scientific Research (TNO), Human Machine Teaming Department, Amsterdam, Netherlands; ^4^Hogeschool Utrecht (HU University of Applied Sciences Utrecht), Utrecht, Netherlands; ^5^Tilburg School of Humanities and Digital Sciences, Department of Communication and Cognition, Tilburg University, Tilburg, Netherlands

**Keywords:** conversational AI, human-AI communication, NLP, chatbots, dialogue systems

The start of 2023 witnessed a disruptive development in Conversational AI: ChatGPT. Large language model technology suddenly became available to millions of users. The underlying GPT-3.5 language model, with 175 billion parameters, trained on 300 billion words and finetuned with human feedback, displayed baffling fluidity, style transfer, and emergent behavior like chain-of-thought reasoning. Moreover, its context window of thousands of tokens enabled a form of conversational training: on-the-fly supervised (albeit volatile) training through prompting. From a conversational perspective, ChatGPT has session-spanning conversational memory, enabling it to pick up on previous interactions in a dialogue. In March 2023, GPT-3.5 was succeeded by GPT-4, with a larger context window, reportedly better accuracy in handling factual questions, and connecting image analysis to language model-based communicative interaction.

Given these significant developments, one could be tempted to think that Conversational AI has come of age. Yet, a full slate of unresolved problems and research questions remains. Crucial debates surround the societal impact of large language models and the future of NLP, the environmental impact of training regimes as well as mass adoption, the impact and prevention of bias, and possible copyright infringement of training data. Central Research Topics in the field of Conversational AI are to a large extent orthogonal to the underlying technology, including large language models. This Research Topic of Frontiers addresses a number of such topics: the human perception of conversational agents and the effects of social cues exhibited by conversational agents on humans, the role of information presentation in hybrid conversational systems, the usage of carefully annotated data in addition to raw textual observational data, and the emergence of communicative patterns between humans and machines.

The paper by Blomsma et al. addresses the perception by human interlocutors of personality traits displayed by embodied conversational agents. These authors demonstrate through a comparison of human-human and human-AI interaction that dynamic social feedback cues, in particular head nodding, correlate with human-perceived personality traits. With Conversational AI becoming increasing multimodal and embedded, these findings will be of practical interest to industry, and may contribute to more natural interaction modes between humans and AI.

The paper of Wieland et al. ties in with these results—these authors investigate the applicability of chatbots for the generation of ideas through brainstorming, taking into account static social cues (name, identity, picture) presented by the chatbot. They find that brainstorming with a chatbot enables participants to generate more and more diverse ideas than through brainstorming with a human, and that the presence of static social cues further reinforces this process. Adding dynamic social cues like the ones identified by Blomsma et al. could actually be an interesting next step.

The paper of Kamoen and Liebrecht also relates to the topic of conversational agent representation and its effect on user interaction. Addressing the topic of Voting Advice Applications (VAAs)- systems that inform humans about political parties and programs during election times-, these authors first assess that chatbot-assisted VAAs lead to higher user satisfaction, and that a hybrid setup (providing users with additional information through informative buttons in a structured user interface) leads to higher user satisfaction, as opposed to pure textual interaction modes. This finding has potential ramifications for the design of task-oriented conversational agents and raises interesting questions about supplementary information presentation modes.

The paper by Bunt and Petukhova underlines the importance of carefully annotated conversational data in addition to raw textual data for finetuning and training conversational AI models.

The authors show that the pragmatic and semantic precision performance of conversational agents benefits from (ISO standardized) formal representations of semantic content, domain-specific communicative functions, and explicit representations of affect. The paper further demonstrates how such annotated data can be used to automatically generate dialogue data in simulation scenarios. A possible implementation of this idea in the current context of Large Language Models would be to impose semantic and pragmatic constraints in annotated data onto a language model through relevance feedback learning, in the same spirit as the human feedback-based reinforcement learning that was applied to finetune ChatGPT.

Finally, the paper by Kouwenhoven et al. addresses the topic of *emergence* in conversational AI: the spontaneous appearance of qualitative traits from the interplay of certain factors (and, for that matter, actors) in communicative settings. Specifically, this position paper focuses on the emergence of grounded shared vocabularies between humans and machines, drawing on insights from language evolution, and pleading for human-assisted reinforcement learning for implementing the preconditions for such emergence.

In summary, this collection of papers addresses important Research Topics on the intersection of humans and conversational agents that eventually will help shape, evaluate and deploy next generations of conversational AI systems.

## Author contributions

SR wrote the initial text. EK, AC, and MW delivered comments, corrections, and reformulations. All authors contributed to the article and approved the submitted version.

